# Adhesional Small Bowel Obstruction at Term: Unmasking a Rare Surgical Emergency in Late Pregnancy

**DOI:** 10.7759/cureus.110947

**Published:** 2026-06-16

**Authors:** Khalisah Shoaib, Osahon Oghodo, Mihraban Bapir

**Affiliations:** 1 Obstetrics and Gynaecology, North Tees and Hartlepool NHS Foundation Trust, Stockton-on-Tees, GBR

**Keywords:** acute abdomen, acute pain in pregnancy, pregnancy acute abdomen, small-bowel obstruction, third trimester complications

## Abstract

Small bowel obstruction (SBO) in pregnancy is an uncommon but potentially life-threatening condition, most commonly caused by adhesions. Its presentation often overlaps with benign gastrointestinal symptoms of pregnancy, making diagnosis challenging and potentially delaying intervention. We report a 38-week primigravida who presented with upper abdominal pain, vomiting, and reduced foetal movements. She was haemodynamically stable with normal laboratory investigations, and MRI demonstrated features of distal small bowel obstruction without a clear transition point. Given the gestational age and persistent symptoms, a caesarean section was performed. Intraoperatively, a fibrous band adhesion across the terminal ileum, causing distal obstruction, was identified, clipped, and divided through the caesarean incision. Both mother and neonate recovered well, and postoperative recovery was uneventful.

This case highlights the diagnostic difficulty of SBO in pregnancy and supports the role of MRI as a safe and effective imaging modality. It also demonstrates the value of multidisciplinary coordination between obstetricians, surgeons, and anaesthetists in optimising outcomes. In selected term patients, addressing obstructing adhesions at the time of caesarean delivery may avoid a separate laparotomy, thereby reducing operative morbidity and facilitating faster recovery.

## Introduction

Small bowel obstruction (SBO) is a rare but important cause of acute abdomen in pregnancy, with an estimated incidence of approximately one in 17,000 pregnancies. Adhesions from previous abdominal surgery are the leading cause, accounting for 50% to 70% of cases [1]. Small bowel obstruction in pregnancy carries significant maternal and foetal risk. Reported maternal mortality is approximately 2%, with complications including bowel perforation (14%), hypovolaemic shock (12%), and sepsis (8%) [2]. Perinatal outcomes are also affected, with prematurity in 45% of cases, low birth weight, stillbirth in 9%, and neonatal death in 4% [3]. 

Physiological and anatomical changes during pregnancy commonly lead to benign gastrointestinal symptoms such as nausea, vomiting, constipation, indigestion, and non-specific abdominal discomfort. These overlaps can complicate the assessment of an acute abdomen and create hesitation in diagnosis and management, particularly when considering both obstetric and foetal factors. Obstetric differential diagnoses for acute abdomen vary with gestational age, ranging from ectopic pregnancy in the first trimester to potential placental abruption in the third, whilst non-obstetric causes of abdominal pain are less frequently considered [4], which adds to the diagnostic challenge and risk of delayed diagnoses of potentially life-threatening non-obstetric emergencies. 

Here, we present a case of a 38-week primigravida, previously fit and well, with a past surgical history of appendicectomy, which predisposed her to the development of adhesions causing the SBO. She presented with generalised abdominal pain, vomiting, and reduced foetal movements. This case highlights the importance of considering surgical causes in the differential diagnosis of abdominal pain in late pregnancy, particularly in women with a history of previous abdominal surgery. It also emphasises the role of imaging modalities, such as MRI, in facilitating timely diagnosis while minimising radiation exposure to both mother and foetus. Early use of appropriate imaging in patients with previous abdominal surgery may help avoid delays in diagnosis and reduce the risk of serious maternal and foetal morbidity and mortality. 

## Case presentation

A 30-year-old primiparous woman presented to North Tees Hospital at 38+1 weeks' gestation with a one-day history of sudden onset epigastric pain, multiple episodes of vomiting, anorexia and reduced foetal movements. Prior to the onset of symptoms, she had eaten pizza the evening before and denied any urinary or bowel symptoms or fever. She noted she had opened her bowels a day before admission and did not complain of constipation. She was previously fit and well, with a past surgical history consisting of an open appendicectomy in childhood. The current pregnancy had been notable for slow foetal growth, from the 50th to the 30th centile; however, her antenatal course was otherwise uncomplicated. She was booked for induction of labour at 39 weeks under midwifery-led care. 

On examination, she was haemodynamically stable and afebrile. Her abdomen was distended in keeping with a term pregnancy, with generalised tenderness, localised in the epigastric region and guarding. Overnight, she had intermittently elevated systolic blood pressure readings, thought to be secondary to pain. Laboratory investigations, including liver function tests, amylase, and infection markers, were within normal limits. Her liquor volume and Doppler were normal; in view of her reduced foetal movements, she was on six-hourly cardiotocography (CTG) as per local guidelines, which were all revealed to be normal. 

An abdominal MRI demonstrated features consistent with SBO, without a distinct transition point. A surgical review was obtained. Based on imaging and clinical findings, the obstruction was initially considered functional, and no immediate surgical intervention was advised. 

Due to the persistent and uncontrolled gastrointestinal symptoms, as well as the MRI findings, the patient consented to a caesarean section in view of findings clinically correlating with a differential diagnosis of SBO at term. During the procedure, the on-call surgical consultant was notified and asked to be present, during which the surgical team re-reviewed the MRI (Figures 1-2) and noted that the imaging demonstrated a distal SBO, likely adhesional in nature, considering the patient’s history of appendicectomy. 

**Figure 1:**
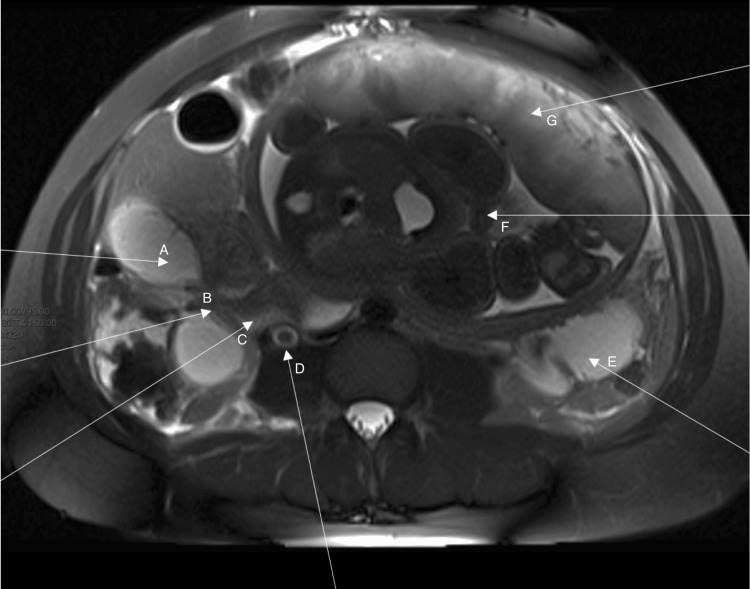
Transverse view of abdominal MRI suggestive of SBO A: Afferent dilated bowel loop, B: Point of transition, C: Distal collapsed bowel loop, D: Right hydroureter, E: Dilated small bowel loop, F: Foetus, E: Placenta; SBO:  Small bowel obstruction

**Figure 2:**
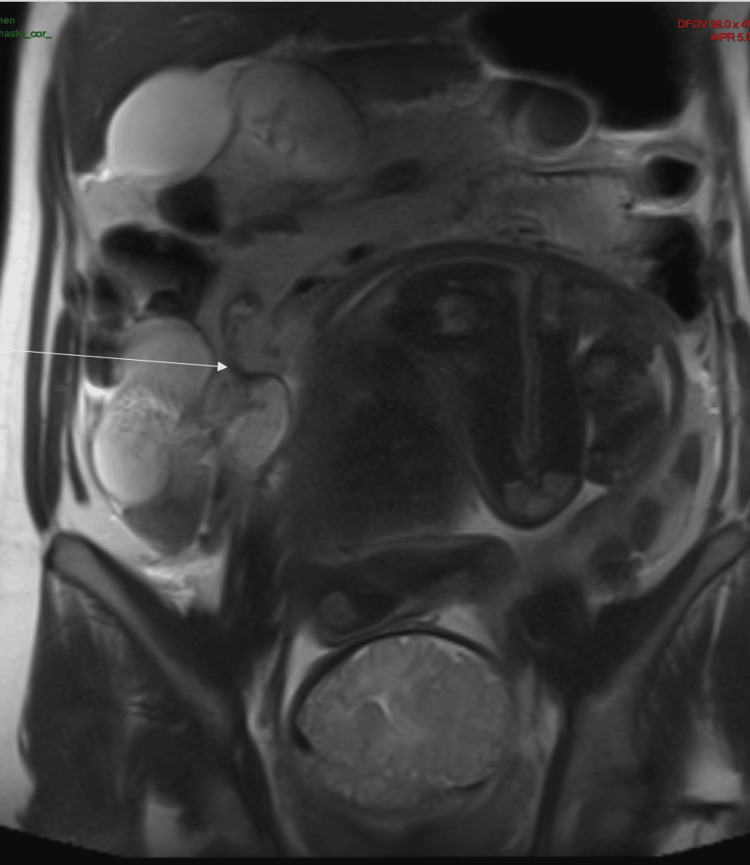
Coronal view of abdominal MRI showing point of transition The arrow points to the single point of transition that caused mechanical blockage of intestinal contents, leading to the clinical presentation described.

Following delivery of a healthy neonate and closure of the uterus, inspection of the abdominal viscera revealed dilated proximal small bowel loops and collapsed distal ileal loops near the caecum. Further exploration under general anaesthesia identified a band adhesion across the terminal ileum, causing a clear point of obstruction, which was released through the low transverse incision (Figure 3). There was no evidence of bowel injury or ischaemia, and no further surgical intervention was required. 

**Figure 3:**
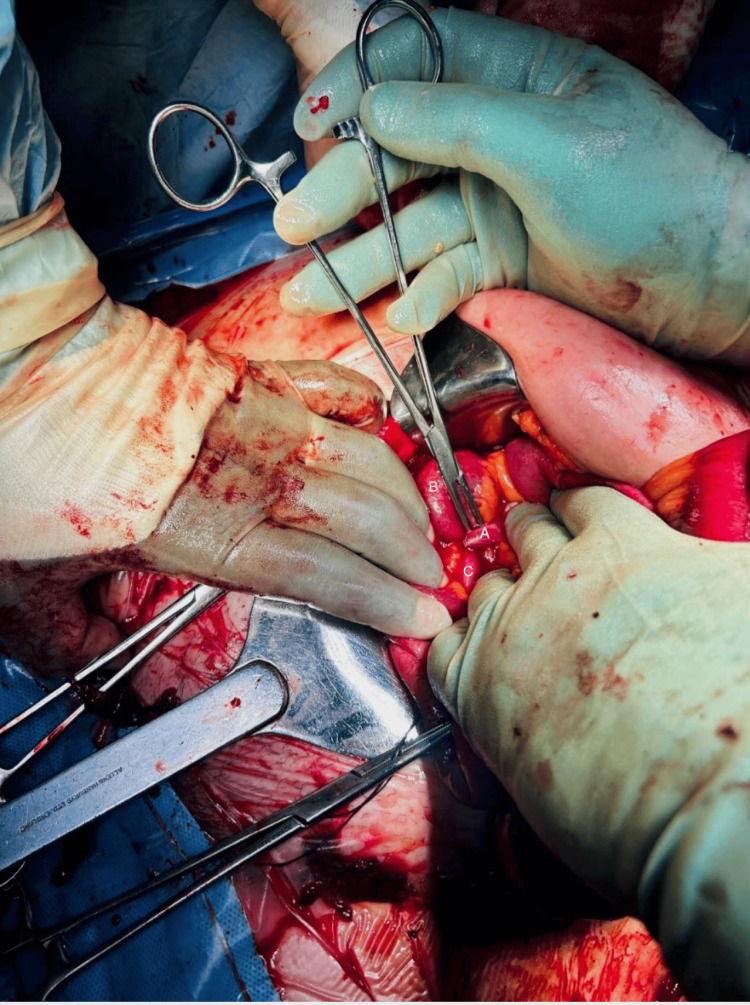
Intraoperative view of the adhesion causing mechanical obstruction A: Adhesion across the terminal ileum, B: Dilated bowel loop, C: Collapsed bowel loop

The patient recovered well postoperatively. Bowel function returned to normal within 48 hours, and both mother and baby were discharged home in good health. The patient was followed up approximately three months following delivery in an outpatient clinic appointment, where she was debriefed again and reported being well and having not encountered any complications.

## Discussion

Small bowel obstruction in pregnancy is a rare condition; however, when it does occur, it is often observed in women with a history of previous abdominal surgery, with postoperative adhesions representing the most common underlying cause [3]. Diagnosis is frequently delayed due to symptom overlap with common gastrointestinal complaints of pregnancy, as well as atypical presentations due to pregnancy, emphasising the importance of maintaining a high clinical index of suspicion. 

The common presentation for classical SBO is colicky central or peri-umbilical abdominal pain, vomiting, abdominal distention, constipation and the absence of flatus [5]. Interpretation of these classical features can be challenging in pregnancy, where the gravid uterus may obscure clinical signs such as distension and alter the perceived localisation of pain. In our patient, the absence of preceding constipation and the passage of both stool and flatus on the day of symptom onset further contributed to diagnostic uncertainty.

In this case, the 33-year-old patient presented in her third trimester with a one-day history of sudden onset, severe epigastric pain associated with persistent vomiting. Clinical assessment demonstrated features concerning for an acute surgical abdomen, including epigastric rebound tenderness, guarding, percussion tenderness involving both upper quadrants, and peritonism. Initial lab investigations showed a mild inflammatory response (white cell counts (WCC) 14.4 × 10⁹/L, neutrophils 11.8 x 10⁹/L), which improved the following day when repeated (WCC 11.9 × 10⁹/L, neutrophils 10.2 x 10⁹/L) with otherwise unremarkable inflammatory markers and biochemistry (CRP 2.7 mg/L, amylase 90 U/L). Abdominal ultrasound identified free fluid within the upper abdomen, but no definitive pathology. Due to the patients' persistent symptoms and concerning examination findings, a surgical review was sought on day two of admission. 

Following surgical review, initial differential diagnoses included upper gastrointestinal perforation due to the severity of abdominal signs and no issues with bowels reported by the patient, having passed bowel motions the day the pain began. However, an erect chest X-ray did not demonstrate free air beneath the diaphragm. Ongoing symptoms and the presence of free fluid prompted further multidisciplinary discussion and MRI evaluation. The MRI findings were described more specifically as mild dilatation of the mid-to-distal small bowel loops measuring >3 cm, with associated free intraperitoneal fluid, collapse of the terminal ileum, and multiple calibre transition points within the right lower quadrant, raising concerns of an SBO. Although the MRI did not identify the exact aetiology of the obstruction, it raised suspicion for a diagnosis that may otherwise have been overlooked or delayed. As demonstrated in the literature, delayed diagnosis of small bowel obstruction in pregnancy can be associated with poorer maternal and foetal outcomes. In this rare and diagnostically challenging case, the MRI played a significant role in reducing clinical uncertainty, guiding further investigation, and expediting appropriate management. 

Imaging modalities, including ultrasound, CT and MRI, each have a role in the assessment of a patient; however, MRI offers advantages during pregnancy due to the absence of ionising radiation and superior soft tissue characterisation [6]. In our case, while abdominal ultrasound and chest radiography were useful adjuncts, MRI provided the most clinically relevant findings and ultimately guided subsequent management. Nevertheless, imaging should always be interpreted within the wider clinical context, as radiological investigations may not always identify the underlying cause of obstruction. 

Management of SBO in pregnancy depends on the suspected aetiology, gestational age, maternal condition and foetal status. Conservative measures, including bowel decompression using a Foley's catheter and close observation, may be considered in clinically stable patients where other differentials have been excluded. However, existing literature remains limited, making definitive conclusions regarding comparative success rates difficult, with one systematic review reporting conservative management is only successful in 7.4% of cases and a failure rate requiring surgical intervention in 10.1% of cases [7]. Similarly, although minimally invasive approaches such as laparoscopic surgery have increasingly been reported across pregnancy, evidence supporting the safety and feasibility of laparoscopy should be interpreted cautiously and individualised according to surgical expertise, gestation and patient factors [8]. Where laparoscopy is not feasible or available, laparotomy may be considered as another viable option.

In this case, persistent symptoms, examination findings in keeping with an acute surgical abdomen, MRI imaging suggestive of SBO, and advanced gestation prompted multidisciplinary discussion regarding operative management. Following joint obstetric and surgical review, and after consideration of maternal preferences, a caesarean section was undertaken with both obstetric and surgical teams present on day three of admission. During the caesarean section, following delivery of the neonate and uterine closure, the transverse incision was extended, and the bowel was inspected. The patient was converted from spinal to general anaesthesia as she was unable to tolerate it. A band adhesion was identified across the terminal ileum, and adhesiolysis was undertaken.

The patient was followed up approximately three months following delivery with no complications noted, and both patient and infant remained in good health. This approach avoided the need for a separate abdominal procedure and enabled definitive management of both obstetric and surgical pathology within a single operation. While such an approach may reduce operative burden in selected patients, further research is required before it is viewed as universally applicable and requires careful individualised assessment of each patient. Our patient was debriefed after one week, followed up at the outpatient clinic six weeks post-delivery, and was seen again at three months as an outpatient. She recovered well with no complications to note.

A key limitation of this study is its single-case design, which limits the generalisability of the findings to broader obstetric populations presenting with SBO. Although the MRI provided valuable diagnostic information, it did not identify the exact aetiology of obstruction pre-operatively, with the definitive cause confirmed intraoperatively, reflecting a limitation in imaging of adhesional SBO. In addition, some radiological parameters, such as bowel calibre measurements and features of ischaemia or closed-loop obstruction, were not fully characterised in the reporting, which may limit the reproducibility of interpretation. The retrospective and descriptive nature of the report also introduces the potential for incomplete data capture and limits the ability to draw causal inferences, particularly regarding long-term outcomes beyond the acute postoperative period. The decision to proceed with combined caesarean section and adhesiolysis was influenced by patient preference and clinical context, introducing an element of selection bias and limiting the extent to which this approach can be considered generalisable. 

This case highlights the importance of considering surgical pathology in pregnant patients presenting with persistent abdominal pain and vomiting, particularly in those with previous abdominal surgery. Early multidisciplinary collaboration between obstetric, surgical and radiological teams remains central to timely diagnosis and optimising maternal and foetal outcomes. 

## Conclusions

Small bowel obstruction in pregnancy is an uncommon but potentially serious condition. This case report reinforces the importance of having a high index of suspicion in pregnant patients presenting with persistent vomiting and abdominal pain, particularly in patients with a history of prior abdominal surgery. Early recognition, prompt imaging, and multidisciplinary involvement are critical to optimising maternal and foetal outcomes. 

Furthermore, in select cases, performing adhesiolysis through the caesarean section incision may be considered a safe and effective approach. This strategy may avoid the need for a separate laparotomy or laparoscopy, reduce operative time, and limit the risk of further adhesions and complications in unstable patients. However, this approach is patient-specific and may not be suitable in all scenarios, particularly when adhesions are complex or bowel compromise is present. 
